# Mapping the translational science policy ‘valley of death’

**DOI:** 10.1186/2001-1326-2-14

**Published:** 2013-07-27

**Authors:** Eric M Meslin, Alessandro Blasimme, Anne Cambon-Thomsen

**Affiliations:** 1 Indiana University Center for Bioethics, 410 West 10th Street, 46202 Indianapolis, USA; 2 UMR 1027, Inserm, Université Toulouse III - Paul Sabatier, 31000 Toulouse, France

**Keywords:** Science policy, Ethics, Valley of death, Stem cell research

## Abstract

Translating the knowledge from biomedical science into clinical applications that help patients has been compared to crossing a valley of death because of the many issues that separate the bench from the bedside and threaten to stall progress. But translation is also inhibited by a science policy environment with its own impediments. Mapping these policy impediments give a more complete picture of the valley of death. Stem cell science is one example where success in moving from the bench to the bedside has confronted policy challenges generating difficulties as challenging as those facing scientists and clinicians. We highlight some of the characteristics and challenges of the science policy valley of death common to the U.S. and Europe, illustrate them with a recent example from stem cell science, and describe some promising strategies for traversing the valley.

## The research and policy ‘valleys of death’

In biomedicine a translational paradigm has been recently adopted to accelerate the pace of scientific progress [1] by focusing attention on the bottlenecks that constitute the so-called “valley of death”, a place in which promising basic research findings go that fail to make their way into (or out of) clinical trials and therefore never have a chance to develop into therapies for patients. But progress in translational medicine is also shaped by science policy, a broad term referring to all efforts to apply science for the benefit of society whether by the public or private sector [2]. We believe that science policy faces its own valley of death, but that comparatively little attention has been given to understanding it in the era of globalized, translational science. We here address the analogy between translational research and science policy development: the detrimental effect of inadequate science policy on translational science and: possible ways of improving the science policy process.

**Figure 1:**
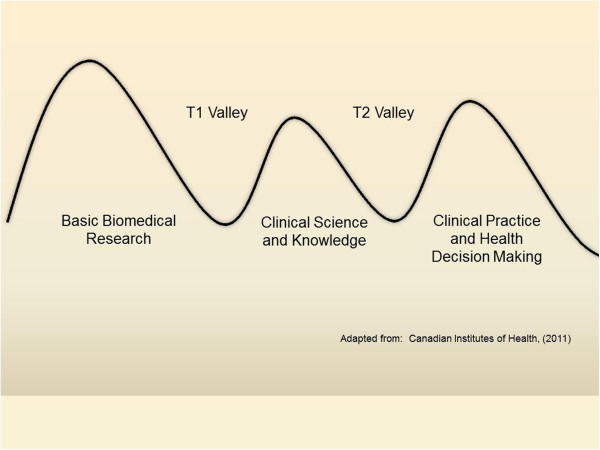
Three hills and two translational valleys from basic research to clinical practice.

**Figure 2:**
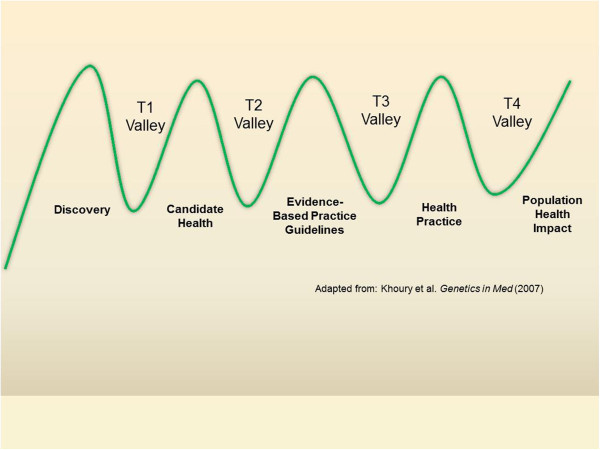
Five hills and four translational valleys from discovery to population health.

Various images are used to portray the bench-to-bedside valley: among the first was of a narrow chasm separating two rocky cliffs. But as translational science evolved so too has the image of this valley topography: rather than one valley, two distinct valleys emerged, the first separating basic research from clinical science, and a second separating clinical science from clinical practice and health decision making [3]. The two valleys have now morphed into five phases of translational research separating four valleys: basic discovery science to research involving humans (T1), from human studies to evidence-based guidelines (T2), from guidelines to health practice (T3), and from health practices to population health programs (T4) [4]. These alternative versions of the valley of death metaphor are summarized in Figures 1 and 2.

**Figure 3:**
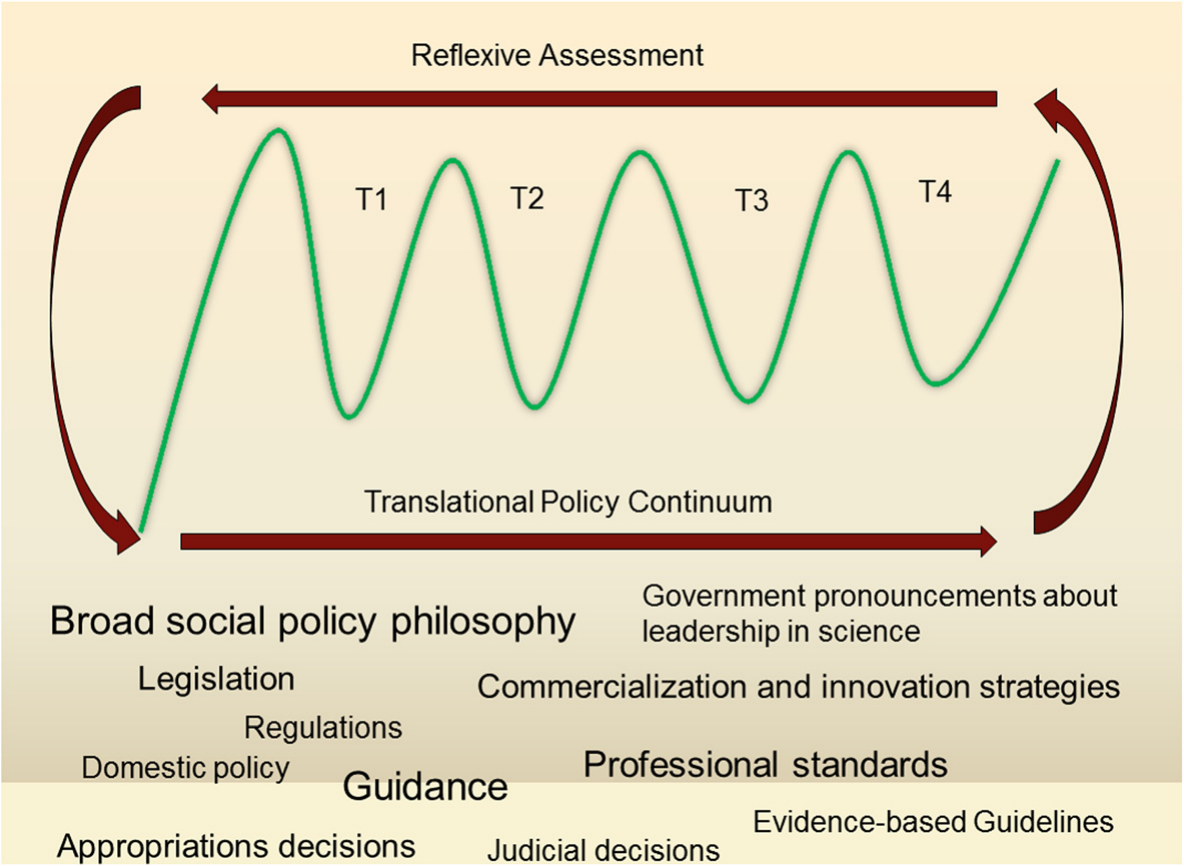
Policy instruments in the translational policy continuum.

The valley metaphor helps explain the impediments that prevent biomedical science from realizing its potential and the risks of failing to translate knowledge into public benefit. The impediments range from lack of technological innovation or “old style thinking” [1], to particular obstacles such as a lack of access to well characterized biological materials in biobanks [5], or insufficient training in translational science for the next generation of investigators [6]. The principal risks of failing to translate this science are the perceived delays in providing diagnostics and clinical care to patients [7,8]. Overcoming these challenges was a key justification for developing translational platforms such as the National Institutes of Health (NIH) Clinical Translational Science Awards (CTSA) program, the Food and Drug Administration (FDA) Critical Path Initiative, the European Advanced Translational Research InfraStructure (EATRIS) in Medicine program, public-private partnership programs like the Innovative Medicines Initiative in Europe, and similar private sector initiatives. All emphasize closer collaboration between investigators, institutions, communities, and sponsors throughout the research lifecycle. Progress in this domain has been slow but steady. Benchmark criteria for achieving success in translational science have been developed [9] and reports of success are being made [10,11]. But sustained progress in biomedical science may depend as much on successfully navigating the dangers of the policy valley as on successfully accelerating translational science itself. On one side of the policy valley we may find broad normative visions about how society might benefit from science progress, innovation and technology development; on the way across the valley lies many varied and pragmatic instantiations of those diverse visions including appropriation decisions, legislation, regulations, guidance documents and other policy instruments for the governance of science and medicine along a translational science continuum. Figure 3 shows the many policy instruments and how they map onto the many valleys illustrated in Figure 2.

Getting from one side to the other involves navigating through a diverse collection of organizations and institutional players – government, private sector and lobbyists among them. Particular challenges include ambiguous regulation [12], unnecessary bureaucracy [13], lack of commercial incentives to innovate [14], and few opportunities to revise legislation or to change habits or practices in the light of new knowledge. Collectively these items stifle the formation of a favorable regulatory environment for innovation, just as readily as does the lack of access to biospecimens, informatics expertise, or nimble ethics review.

Science policy implies differences in focus, needs and strategy [15] which, if not addressed, may severely hamper the efficiency of the translational process. Consider funding as an example. In an 1864 letter to the French Ministry of Public Instruction, Louis Pasteur requested additional funding for his fermentation research by underlining its importance for the French wine business [16]. This historical example reminds us that science funding is one of the most visible (and vigorous) challenges in science policy. Indeed, by most accounts the billions of dollars spent to increase the NIH budget and for similar commitments to “frontier research” by the European Union have been well spent from the perspective of both the basic and applied sciences. But if deployed poorly, science policy can do the opposite: it can stifle the creative pursuit of knowledge, bar access to treatments by inhibiting the approval process for medicines [17] or discourage scientists from pursuing promising avenues of inquiry [18]. One may look no further than the fiscal cliff off of which U.S. science funding has plummeted, or the comparable reductions in Europe’s proposed Horizon 2020 budget to find examples of how science policy funding decisions create risks for science. The impact of a $2.5 Billion cut from the US science budget is now trickling down to every public university. In Europe the impact on young investigators would be especially dire and will have a profound impact on society [19].

While fiscal cliffs and funding decisions rightly occupy political attention, and are often held hostage to political ideology, some science policy involves legitimately controversial ethical topics for which a policy pathway across the valley would be welcomed. Below we consider the case of stem cell research, especially embryonic stem cell research, as an example whose history demonstrates how success in crossing the biomedical science valley may be hampered by an inadequate policy map. Other examples could also illustrate the process, such as gene patenting, use of existing biological samples for research, data sharing according to funding used for research.

## Embryonic stem cell science and stem cell policy

Embryonic stem cell science may optimistically be characterized as moving across the T1 valley from basic discoveries to initial applications in health – though even recent developments, such as the report of the first cloned human stem cells [20], remind us that basic science work continues in this field. There was a time not long ago, however, when it might not have been pursued at all. Beginning with the momentous announcement in early November 1998 that human embryonic stem cells had been isolated [21] strategies for translation were hindered by numerous ethical and political hurdles. Even early claims of success turned out to be either unsupported [22] or patently false [23]. Science persisted with successes reported in other areas of stem cell science, among them Yamanaka’s Nobel prize-winning work on induced pluripotent stem cells [24]. Arguably, however, these advancements may have occurred despite the policy environment rather than because of it. For example, in the immediate aftermath of the 1998 reports U.S. President Clinton requested that the National Bioethics Advisory Commission advise on next steps [25] which was followed by actions from the NIH, FDA, the Institute of Medicine, Congress and two presidents to craft U.S. domestic federal policy that is still somewhat unsettled. This was despite more than two decades of policy deliberation in the US about ethics of embryo research. Many other jurisdictions including those in Europe developed evolving policy in parallel with the U.S. For example, France’s first bioethics law in 1994 prohibited research on embryo stem cells and evolved over time: following revisions of the law in 2004 certain exceptions to the prohibition were permitted, then a revision of these conditions were described in 2011, and most recently in 2013 ES cell research was proposed to be authorized with conditions. Others established country-specific rules ranging from permissive, albeit highly regulated regimes, to restrictive laws forbidding the isolation of stem cells from human embryos under any circumstances [26]. The policy landscape at that time included no shortage of recommendations, regulations, guidelines and legislation but rather than offering a clear path for translational medicine they were often unclear, ambiguous and sometimes contradictory. In some instances the uncertainty was shown to have demonstrable (and substantially detrimental) effects on the conduct of stem cell science as reported by scientists themselves [18]. This ‘patchwork’ approach was found in other countries leading to frustration and confusion by scientists and sponsors [27].

While some efforts to reduce uncertainty have been achieved [28], progress across the policy valley still faces impediments as two recent examples illustrate. In August 2012, the US district court for the District of Columbia issued a landmark decision (*USA v*. *Regenerative Sciences*) confirming that the FDA has jurisdiction over the commercialization of autologous mesenchymal stem cell therapies [29]. Later the same month, an Italian judge took the opposite position authorizing heterologous injections of mesenchymal stem cells in a two year-old girl affected by spinal muscular atrophy, a condition that is currently incurable [30]. The procedure was conceived and already undertaken on a number of patients by a physician collaborating with a not-for-profit organization called Stamina Foundation. Their method, however, was not supported by any published pre-clinical or clinical data about the safety and efficacy of the procedure. For this reason, AIFA – the public agency that oversees medical drugs in Italy – had initially prohibited Stamina from carrying out the procedure on patients. Once the Italian judge’s decision established a precedent, identical ones followed suit as a growing number of families sought to continue or access the same procedure for their children.

What is particularly troubling is that the alleged treatment was administered under a so-called compassionate use protocol, which is tightly regulated by Italian legislation. It seems, however, that this did not prevent the judges from interpreting the law in a way that overlooked two crucial legal requirements for compassionate use of unproven cellular therapy: first, the existence of published scientific evidence justifying the procedure and, second, a favorable risk-benefit assessment by a local ethics committee.

The case did not escape the attention of the scientific community that roundly condemned the Stamina procedure and the permissive attitude of the Italian judges [31,32]. In a statement from the International Society for Stem Cell Research (ISSCR), Yamanaka bemoaned the Italian decision, stating that “stem cell therapy and treatment decisions should not be made outside of a controlled clinical trial with data on safety and efficacy” [33].

Notwithstanding their obvious shortcomings a mounting wave of public support for an alleged right to access those unproven infusions, pushed the Ministry of Health to issue an *ad hoc* executive decree. The latter was recently amended and approved by the Italian Parliament and it is based on two controversial elements: first, for the next eighteen months the Stamina method, albeit unproven, will be freely available to patients affected by rare diseases; second, the State will allocate three million Euros to sponsor a clinical trial designed to test the safety and efficacy of the Stamina procedure.

The Italian judicial decision and the Parliamentary debate that brought to the ratification of the ministerial decree are clear examples of the messiness of the policy process and the dangerous consequences of inadequate policy support. But the Italian policy steps are also illustrative of an important shift of emphasis: the moral status of the embryo which for years dominated the debates about the permissibility of embryonic stem cell research is no longer the main impediment to the progress of stem cell science. Rather, policy may now be the principal impediment to its sustained progress, especially when it may be used to support dubious hopes and permit use of public resources for purposes that lack evidence. There is a lesson here: as science moves into controversial areas, public expectations will have to play a role in shaping the direction of research and innovation; however, we must be wary of potentially unreasonable demands on the part of the public with respect to scientific innovation. As the Italian case seems to demonstrate, a better understanding is required of the right balance between scientific promises and attainable futures, and of the proper relationship between expert communities, decision makers and the diverse publics affected by science policy decisions.

### Crossing the policy valley

Several promising areas are emerging that indicate ways the policy valley can be more effectively crossed. Here we highlight four of them.

### Renegotiate the science social contract with society

For more than six decades many countries sought to implement their own version of Vannevar Bush’s “endless frontier” in which science would not only provide solutions to health problems but enhance wealth and prosperity [34]. The structuring of universities to engage in teaching and research, the establishment of major biomedical research organizations, and the dramatic increases in public funding for science were hallmarks of the second half of the 20^th^ century’s “frontier”. In many countries in Europe as well as in USA these were the policy acts of an implied social contract the terms of which were straightforward: in exchange for public support, the scientific community would be granted substantial autonomy to conduct research with the expectation that the products of their investigations would be returned to society in the form of knowledge and applied technology [35]. Generally speaking, like the social contract theory developed by Hobbes, Locke, Hume, and Rousseau, which described the optimal relationships between individuals and government, society’s contract with science was also intended to function for the reciprocal benefit of both. This has been particularly so for “big science” projects which promote social cohesion or other political goals such as Europe’s decision to build CERN, President Kennedy’s push for the moon, or the Human Genome Project. It has also been a common justification for translational research generally [36].

While the metaphorical social contract may have functioned for several decades, in recent years calls to radically update or retire it completely have grown [37]. Even if we undertake a thorough re-thinking of the way society ought to interact with science [38] it is unlikely that a renewed social contract alone will be sufficient to deal with the policy challenges arising in stem cell science or other controversial topics. Not only does the format of science-society interactions need re-adjustment but so too does the very positioning of these activities in the research and policy decision processes. A recent European Science Foundation (ESF) report indicates that the classical social contract for science is no longer operational and proposes several recommendations, two of which we would like to underline as they are of immediate relevance to the issues addressed here [39]. First, the ESF calls for “expanding and creating new spaces for science-society interactions” insisting on bottom-up initiatives and creativity in this domain. The expected advantage of effective public engagement in shaping the direction of innovative science is the broadening of the innovation agenda to a wider array of values and interests. Second, the ESF report advocates enhancing the opportunities for reflexive assessment of scientific innovation. Reflexive thinking refers to the capacity to put under critical scrutiny the assumptions, expectations, values and visions of the future that are embedded in specific modalities of producing knowledge and innovation. Those visions shape the pace and direction of science, and drive the ‘competition race’ that characterizes today’s science. However, they are rarely made the object of sustained analytical attention. Instead, we believe that occasions for reflexive assessment of science with respect to broader sets of societal values should be abundant. This means incentivizing research in the humanities and the social sciences about how innovation relies on societal expectations and, at the same time, how societal expectations are determined by scientific novelties. The governance of science has such a pervasive societal impact that it deserves state-of-the-art analytic tools for its assessment and improvement. Furthermore, reflexivity also implies that even the proposed modalities of engagement between science and society are constantly questioned, assessed and, where possible, improved. Research in this domain is thus crucial and this brings us to our next indication.

### Gathering the evidence base for science policy

If we are to be serious about harnessing the potential of science in the interest of society, we need governance initiatives that, rather than prompting ideological confrontation, are able to occasion upstream, reciprocal learning between science and society and thus intelligent, evidence-based decision making about scientific research and its various applications [38,39]. One such initiative with which two of us are associated is called ‘ELSI 2.0’, an innovative international collaboratory that is designed to engage more international collaboration in the ethical, legal and social implications of genomics research and accelerate translation from research to practice [40]. ELSI 2.0 will function by leveraging the cooperative power of many individuals, groups and stakeholders across the globe through shared research platforms, mixed-methods for engagement, and other approaches to accelerate the efficient and effective application of genomics for society. Other promising developments include the evidence-derived research agenda that emerged from 48 science policy experts who recommended 40 key questions to “catalyse and focus research in this field” [41], the ongoing efforts to collect data about policy influences [42], and the recent establishement of a database on global health research in Africa developed by many of the world’s foremost funding bodies [43]. These efforts are necessary steps to empirically understand the impediments to implementing science policy and to combine these with needed ethical reflection. We encourage more efforts to gather empirical data about the possible impact of different governance options of novel science and technology. While we are confident that a more inclusive public dialogue about science and well-designed participatory initiatives are per se promising tools for improving the governance of scientific innovation, it is indispensable that such discussions rely on a solid evidence base to reach beyond academic circles, and to be taken into consideration at the policy level.

### Reframing funding priorities

The public investment in science is too often justified mainly for its capacity to generate jobs and to attain primacy in the geopolitical competition of the global knowledge economy [44-46]. Whereas in the short-term this narrative can provide a good deal of resources for science, in a medium to long-term perspective it can stifle its development and curtail its expectations, should other sectors come to be seen as more appealing for public investment. Science funding, in other words, should be inspired by the value of science itself including its interactions with society and capacity of reflexivity as a very part of scientific activity. Emphasis on derivative aims such as economic development, while important, may risk avoiding the need to critically assess the societal mission of science. Science has so much to offer that it should not be afraid of engaging is such reflexive exercise: quite to the contrary, open dialogue in this domain can revitalize and update its role in society.

### Acknowledge common goals

Science policy rarely proceeds in an orderly fashion. This is because policy construction, development, revision, public input, implementation and evaluation form a complex system undertaken over long periods of time, by multiple actors located in multiple sites who are influenced by contextual, economic and other political factors. Science policy is complex for another reason, in that it is often responsive to unplanned events. Despite these factors there are some hopeful signs for deploying science policy more effectively. Japan’s recent experience in response to its devastating tsunami showed that public trust is a necessary activity for countries seeking to implement science policy-making [47]. This view is consistent with calls to democratize science which will require sustained critical reflection and creative political experimentation [48] in setting up inclusive governance initiative for innovation across a variety of scientific domains. Democratization, however, faces two major challenges. On the one hand, more inclusive governance processes may end up including non-reconcilable visions and values. The Stamina case, for example, raises a number of doubts about how we may be able to reduce the gap between radically diverging framings of regenerative medicine. The second challenge arises from the global environment in which scientific innovation is occurring especially regarding coordinated governance initiatives across a complex environment of cultural, political and social variation.

However, both challenges represent a stimulus for a more sustained interaction between science, the social sciences and the humanities to construct the foundations for tomorrow’s initiatives in international science policy. Returning to the example of embryonic stem cell research, these are still early days of stem cell policy innovation – so it is hard to craft science policy acceptable to society for supporting research and development in the service of the public good when it is difficult to predict where the science and its applications are likely to go. And yet that is precisely the role of science policy. Much effort was expended in the early days of embryonic stem cell research to design appropriate guidelines for laboratory-based activities. These problems have now been replaced by others such the issue of unproven stem cell therapies and stem cell tourism which, arguably, are in greater need of a coordinated approach to global science policy. Both topics can exploit regulatory loopholes and country-specific differences, yet so far, the only kind of international governance action has been taken by self-regulating scientific societies such as the ISSCR [28,49]. In the absence of trusted national authorities to adjudicate on matters of science, or global institutional bodies with enough credibility to speak to these issues, renewed attention should be paid to global governance in stem cell research, as we are now beginning to see for areas such as research involving biobanks [50].

### Summary

Science policy lies at the intersection of science and public policy. Its objects are the proper funding of and priority-setting for the governance of science, the regulation and conduct of research, and all related efforts to apply science for the benefit of society. Science is also a global, interconnected activity and as such the development of science policy in one country implicates citizens, scientists and governments in other countries. As stem cell science illustrates, this landscape is changing quickly. This proves the obvious: that the pace of scientific innovation is fraught with possible controversies, spanning from ethical to societal and legal ones. Therefore, a long-term public commitment in favor of science cannot be decoupled from the necessity to exert reasonable forms of oversight on scientific research and its application. Managing public disagreement about science in a way that can be perceived as legitimate across the broadest possible range of moral perspectives [51] is certainly no easy task. This task should thus inspire more sustained and dedicated efforts at analyzing the impact of science policy options on the course of scientific innovation: we need to engage in reflexive and imaginative thinking about science and science policy implications. Only by giving the same attention to bridging science policy’s valley of death as we do to biomedical research translational process, prospects can be favorable for the effective translation of science into collective benefit.

## Competing interests

At the time of this writing EMM was a consultant to Eli Lilly and Company. AB and ACT declare that they have no competing interests.

## Authors’ contributions

EMM jointly conceived of the original idea for the study and helped to draft the manuscript. AB provided critical new insights for the study, and helped to draft the manuscript. ACT jointly conceived of the original idea for the study, and helped to draft the manuscript. All authors read and approved the final manuscript.

## Authors’ information

Eric M. Meslin, Ph.D., is Director of the Indiana University Center for Bioethics, Associate Dean for Bioethics, Indiana University School of Medicine, Professor of Bioethics, and Director of the Bioethics and Subject Advocacy Program of the Indiana Clinical and Translational Science Institute. He has been involved in bioethics policy for much of his career including directing the ELSI program at NHGRI, and as Executive Director of the National Bioethics Advisory Commission appointed by President Clinton.

Alessandro Blasimme, Ph.D., is a post doctoral researcher at INSERM (French National Institute of Health and Biomedical Research) in joint research unit UMR1027 INSERM – Université Paul Sabatier, Toulouse, France. As a bioethicist, he is involved in two European FP7 projects: CAGEKID (Cancer Genomics of the Kidney), and ESGI (European Sequencing and Genotyping Infrastructure). Thanks to research grant from the Midi-Pyréneés Region he is also conducting research on the ethical and regulatory aspects of the governance of stem cell-based medicine.

Anne Cambon-Thomsen, MD, is Director of Research in CNRS (French national centre for scientific research) working in a research Unit on epidemiology and public health at Inserm (National Institute for Health and Medical Research), and University of Toulouse III Paul Sabatier, Faculty of Medicine Toulouse, France. Specialist in human immunogenetics and active in bioethics since the late 90’s, she leads an interdisciplinary research team on “Genomics, biotherapies and public health”, involving human and social sciences as well as health sciences and the “Genetics and Society” societal platform of the Toulouse-Midi-Pyrénées Genopole. Former member of the CCNE (French national advisory bioethics committee) and of the European Group on ethics of science and new technologies (EGE), and member of the ethics and deontology committee of the National Cancer Institute in France, she worked in recent years on societal aspects of biobanks, biotherapies, transplantation, genetic testing, biomarkers, high throughput technologies, data sharing, and biotechnologies.
